# Mining causal relationships among clinical variables for cancer diagnosis based on Bayesian analysis

**DOI:** 10.1186/s13040-015-0046-4

**Published:** 2015-04-16

**Authors:** LiMin Wang

**Affiliations:** 1 Key Laboratory of Symbolic Computation and Knowledge Engineering of Ministry of Education, JiLin University, ChangChun, 130012 P. R. China; 2 State Key Laboratory of Computer Science, BeiJing, 100080 P. R. China

**Keywords:** Causal relationship, Cancer diagnosis, Restricted Bayesian classifier

## Abstract

**Background:**

Cancer is the second leading cause of death around the world after cardiovascular diseases. Over the past decades, various data mining studies have tried to predict the outcome of cancer. However, only a few reports describe the causal relationships among clinical variables or attributes, which may provide theoretical guidance for cancer diagnosis and therapy. Different restricted Bayesian classifiers have been used to discover information from numerous domains. This research work designed a novel Bayesian learning strategy to predict cause-specific death classes and proposed a graphical structure of key attributes to clarify the implicit relationships implicated in the data set.

**Results:**

The working mechanisms of 3 classical restricted Bayesian classifiers, namely, NB, TAN and KDB, were analysed and summarised. To retain the properties of global optimisation and high-order dependency representation, the proposed learning algorithm, i.e., flexible *K*-dependence Bayesian network (FKBN), applies the greedy search of conditional mutual information space to identify the globally optimal ordering of the attributes and to allow the classifiers to be constructed at arbitrary points (values of *K*) along the attribute dependence spectrum. This method represents the relationships between different attributes by using a directed acyclic graph (DAG) model. A total of 12 data sets were selected from the SEER database and KRBM repository by 10-fold cross-validation for evaluation purposes. The findings revealed that the FKBN model outperformed NB, TAN and KDB.

**Conclusions:**

A Bayesian classifier can graphically describe the conditional dependency among attributes. The proposed algorithm offers a trade-off between probability estimation and network structure complexity. The direct and indirect relationships between the predictive attributes and class variable should be considered simultaneously to achieve global optimisation and high-order dependency representation. By analysing the DAG inferred from the breast cancer data set of the SEER database we divided the attributes into two subgroups, namely, key attributes that should be considered first for cancer diagnosis and those that are independent of each other but are closely related to key attributes. The statistical analysis results clarify some of the causal relationships implicated in the DAG.

## Background

Cancer is the second leading cause of death around the world after cardiovascular diseases. Predicting the outcome of cancer is one of the most interesting and challenging tasks for data mining applications. To realise such a task, medical research groups collect large volumes of medical data and employ computers with automated tools. Thus data mining techniques have become a popular research tool among medical researchers for identifying and exploiting patterns and relationships among numerous variables, interpreting complex diagnostic tests and predicting the outcome of a disease by historical data sets. The rapid progress of data mining research has led to the development of medical diagnostic support systems, which are now extensively applied across a wide range of medical area, such as cancer research, gastroenterology and heart diseases. Pena and Sipper [1] indicated that effective diagnostic systems should provide high-accuracy disease identification. Effective systems should also confidently indicate the accuracy of the diagnosis with some levels. Another major important aspect of competent systems is their interpretability, i.e., providing information on the steps followed to obtain outcomes.

Cancer diagnosis has received considerable attention from researchers, and many classical data mining algorithms have been used in medical data analysis. Decision trees can be easily understood and interpreted for domain experts. This area has been extensively explored for the past few years. Learned trees can be represented as a set of “if-then rules” that improve human readability. C5.0 is one of the most important algorithms in the decision tree family. Rafe et al. [2] used the C5.0 algorithm to develop a model for Clementine software and to form a confusion matrix. The database used for the experimental study was ”Wisconsin Breast Cancer database”, which contains 10 attributes and 699 instances. By using the boosting method, the precision of the final model can be increased to decrease the percentage of error. Khan et al. [3] proposed a hybrid prognostic scheme based on weighted fuzzy decision trees(FDT). Such a scheme is an effective alternative to crisp classifiers that are applied independently. A hybrid prognostic scheme analyses the hybridisation of accuracy and interpretability in terms of fuzzy logic and decision trees. They used the Surveillance Epidemiology and End Results (SEER) database (1973 to 2003) of the National Cancer Institute, which consists of 162,500 records with 17 variables after pre-processing. The resulting AUC values were 0.69 and 0.77 for FDT and weighted FDT, respectively. Carefully designed pre-processing procedures help achieve the removal/modification/splitting of key attributes. Agrawal et al. [4] discovered 2 of the 11 derived attributes that have significant predictive power. These researchers employed the ensemble voting of 5 decision tree-based classifiers and meta-classifiers to acquire the best prediction performance in terms of accuracy and area under the ROC curve. The experimental study was performed on the pre-processed data along with various data mining optimisations and validations.

Artificial neural networks (ANNs) are commonly known as biologically inspired, highly sophisticated analytical techniques that can model extremely complex non-linear functions. ANNs have been proven to be an effective classification tool even in hidden operations within a network structure. Motalleb [5] incorporated a multilayer feed-forward neural network with an error back-propagation algorithm to develop a predictive model. The input parameters of the model were virus dose, week and tamoxifen citrate. Tumour weight was included in the output parameter. Two different training algorithms, namely, quick propagation and and Levenberg-Marquardt, were used to train ANN. To minimize user effort, Vukicevic et al. [6] applied genetic algorithms to achieve the best prognostic performances relevant for clinicians (i.e., correctness, discrimination and calibration). The only 2 user dependent tasks were data selection (input and output variables) and the evaluation of the ANN threshold probability with respect to regret theory (RT). After optimally configuring ANNs with respect to these criteria, the clinical usefulness was evaluated by the RT Decision Curve Analysis. Tsao et al. [7] developed an ANN model to predict prostate cancer pathological staging in patients prior to receiving radical prostatectomy. This experimental study examined the cases of 299 patients undergoing retro-pubic radical prostatectomy. In this investigation, the validation was assessed by using the current Partin Tables for the Taiwanese population. ANN induced larger AUCs and provided a more accurate prediction of the pathologic stage of prostate cancer.

Bayesian networks (BNs) are characterised by the use of the probabilistic approach in solving problems and encompass the uncertainty of specific occurrences. The origin of BNs is based on probability distribution, which can be graphically depicted. Alexander et al. [8] applied the SEER database (1969 to 2006) to form a clinical decision support system for the real-time estimation of the overall survival (OS) rate of colon cancer patients. The BN model accurately estimated OS with an area under the receiver-operating characteristic curve of 0.85. They significantly improved upon the existence of AJCC stage-specific OS estimates. Furthermore, they determined the significant differences in OS between low- and high-risk cohorts. Khan et al. [9] used Bayesian method to derive the posterior density function for the parameters and the predictive inference for future survival times from the exponentiated Weibull model, assuming that the observed breast cancer survival data follow such type of model. The Markov chain Monte Carlo method was used to determine the inference for the parameters. They found that the exponentiated Weibull model fits the male survival data. Mean predictive survival times, 95% predictive intervals, predictive skewness and kurtosis were obtained. Jong et al. [10] introduced a hybrid model that combined ANN and BN to obtain a good estimation of prognosis and a good explanation of the results. In this research, the SEER database (1973 to 2003) was employed to construct and evaluate the proposed models. Nine clinically acceptable variables were selected to be incorporated into the nodes of the proposed models. Consequently, the hybrid model achieved the highest area under the curve value of 0.935, and the corresponding values of ANN and BN were 0.930 and 0.813, respectively.

Other machine learning models have also been applied to solve the problems in predicting cancer survivability. Molina et al. [11] suggested that an incremental learning ensemble of a support vector machine (SVM) must be implemented to adapt to the working conditions in medical applications and to improve the effectiveness and robustness of the system. These studies calculated the probability estimation of cancer structures by using SVM and performed the corresponding optimisation with a heuristic method together with a three-fold cross-validation methodology. Mahmoodian et al. [12] developed a new algorithm on the basis of fuzzy association rule mining to identify fuzzy rules and significant genes. In this study, different subsets of genes that have been selected by different methods were used to separately generate primary fuzzy classifiers. Subsequently, the researchers administered their proposed algorithm to mix the genes associated with the primary classifiers and to generate a novel classifier.

Only a limited number of conditional probabilities can be encoded into BN because of time limitation and space complexity. The restricted BN classifier family can offer different trade-offs between structure complexity and prediction performance. The conditional independence assumption and different levels of extra dependencies between predictive attributes signify that some learning algorithms (e.g., Naive Bayes (NB) [13,14], tree-augmented Naive Bayes (TAN) [15] or *K*-dependence BNs (KDB) [16,17] are popular ever since they were developed both for learning BN parameters and data structures. An optimal Bayesian classifier should capture all or at least the most important dependencies among attributes that exist in a database. In the next section, the working mechanisms of three popular restricted BN classifiers (i.e., NB, TAN and KDB) are summarised. Consequently, the proposed learning algorithm, namely, the flexible *K*-dependence Bayesian network (FKBN), applies greedy search in conditional mutual information (*CMI*) space to maximise the information flow between attributes and to globally describe causal relationships while maintaining high dependency representation. The proposed algorithm also allows the construction of classifiers at arbitrary points (values of *K*) along the attribute dependence spectrum. We compare these classical Bayesian models that predict the survivability of patients diagnosed with breast cancer. In this study, such a prediction is addressed by a classification problem that predicts whether the patient belongs to the group of those who survived after a specified period. We aim to determine an accurate and stable classification model that will allow medical oncologists to make efficient decisions for treating cancer patients.

## Materials and methods

### Data

For this experimental study, 12 data sets are selected and collected to clarify the clinical implications of the causal relationship among clinical variables and to discuss the application of the proposed method to the high-dimensional genomic data. Table 1 summarises the characteristics of each data set, including the numbers of instances, attributes and classes. The first 6 data sets are collected from the SEER database [18], which is a unique, reliable and essential resource for investigating the different aspects of cancer. Moreover, this database combines patient-level information on cancer site, tumour pathology, stage and cause of death. The remaining 6 data sets (e.g., gene expression, protein profiling and genomic sequence that are related to classification) are acquired from the Kent Ridge Bio-Medical (KRBM) repository [19].

**Table 1:** **Data sets for experimental study**

No.	Data set	# Instance	Attribute	Class
1	BREAST	346317	200	3
2	FEMGEN	396386	200	3
3	LYMYLEUK	324441	200	3
4	MALEGEN	552483	200	3
5	COLRECT	477237	200	3
6	URINARY	272646	200	3
7	ALL-AML_Leukemia	38	7130	2
8	DLBCL-Harvard	77	7130	2
9	DLBCL-Stanford	47	4027	2
10	LungCancer-Michigan	96	7130	2
11	MLL_Leukemia	57	12583	3
12	ProstateCancer	102	12601	2

The first six data sets are selected from the SEER database, the next six data sets are selected from Kent Ridge Bio-medical (KRBM) repository.

### Restricted Bayesian classifier

Bayes’ chain rule can be used to form a classifier for an input vector *X*={*X*_1_,⋯,*X*_*n*_} and class variable *C*.

(1) $$ P(c|x)=\frac{P(c)P(x|c)}{P(x)}\propto P(c)P(x|c)=P(c)P(x_{1}|c)P(x_{2}|x_{1},c)\cdots P(x_{n}|c,x_{1},\cdots,x_{n-1})  $$

Where the lower case letters represent the possible values taken by the corresponding attributes. If Eq.(1) is represented by Bayesian model, the attribute vector {*C*,*X*_1_,⋯,*X*_*i*−1_} is considered the parent attribute of *X*_*i*_, i.e., *Pa*_*i*_.

∙ NB (0-dependence classifier). In NB, class label *c*^∗^ will be inferred from a Bayesian model of an *n*-dimensional attribute (input) vector *X*, which is conditionally independent given class variable *C*

(2) $$ c^{*}=\arg\max P(c)P(x|c)=\arg\max P(c)\prod_{i=1}^{n}P(x_{i}|c)  $$

**Figure 1:**
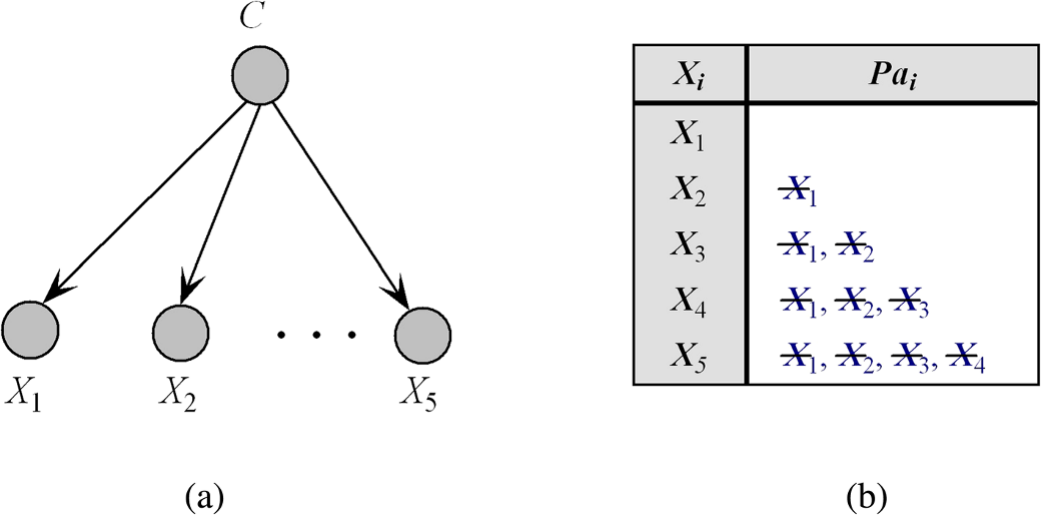
The 0-dependence relationship among attributes of NB model and corresponding *Pa*_*i*_ of each attribute *X*_*i*_ are as **(a)** and **(b)** show, respectively. The attributes annotated with symbol "-" represent those redundant ones for NB. For simplicity, class label is not included in *Pa*_*i*_.

Corresponding belief network is graphically depicted in Figure 1(a). Each predictive attribute node in NB has the class variable as its only parent. Therefore, NB enjoys the benefit of not being required to learn the structure, and probabilities *P*(*c*) and *P*(*x*_*i*_|*c*) can be easily estimated from training instances. Figure 1(b) illustrates that all causal relationships among the predictive attributes are removed; thus, NB is the simplest form of BNs. However, the conditional independence assumption made by NB is rarely valid in reality.

**Figure 2:**
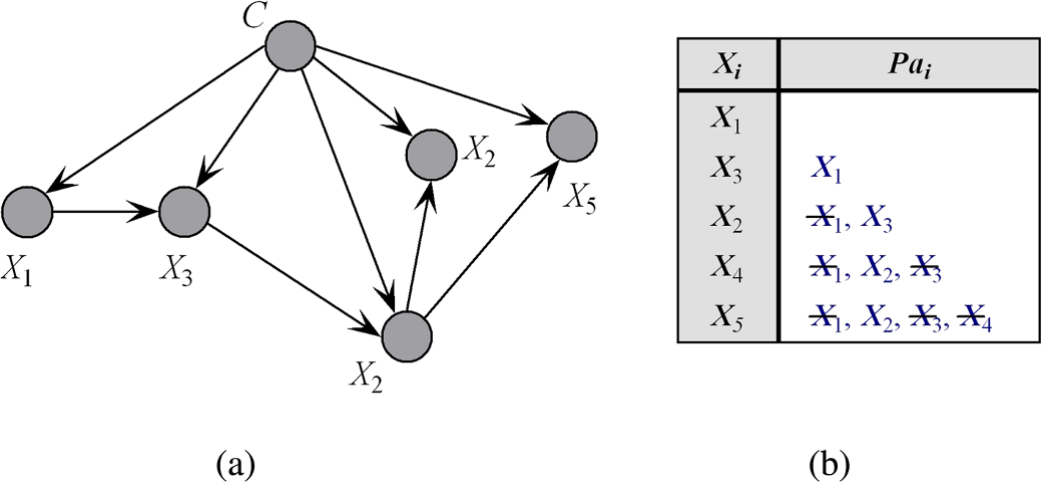
The 1-dependence relationship among attributes of TAN model and corresponding *Pa*_*i*_ of each attribute *X*_*i*_ are as **(a)** and **(b)** show, respectively. The attributes annotated with symbol "-" represent those redundant ones for TAN. For simplicity, class label is not included in *Pa*_*i*_.

∙ TAN (1-dependence classifier). In effectively weakening the conditional independence assumption of NB, structure extension is the most direct procedure for improving NB because attribute dependencies can be explicitly represented by arcs. TAN introduces more dependencies by allowing each attribute node to have at most one parent. An example of the network structure of TAN with five attributes and with corresponding causal relationships are depicted in Figure 2(a) and (b), respectively. By developing a maximum weighted spanning tree, TAN achieves a globally optimal trade-off between the complexity and learnability of the model. However, the number of dependencies that can be represented is limited, and this algorithm cannot be extended to handle high-dependence relationships. The weight of arcs is calculated by using *CMI*(*X*_*i*_,*X*_*j*_|*C*).

**Figure 3:**
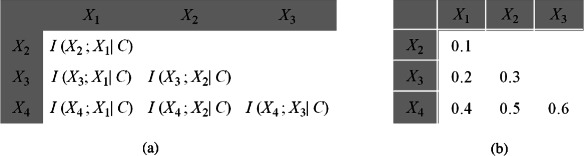
The format and an example of *CMI* matrix are as **(a)** and **(b)** show, respectively. Because of the symmetrical characteristic of conditional mutual information, only the lower triangular matrix is shown.

**Figure 4:**
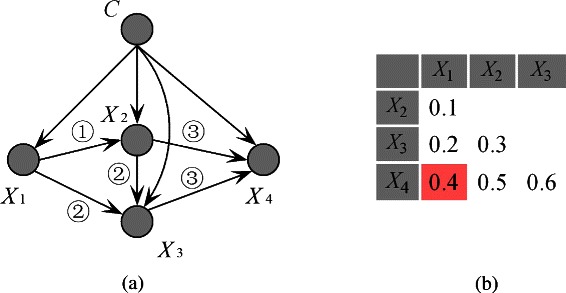
The *K*-dependence relationships among attributes inferred from KDB and an example of *CMI* matrix are as **(a)** and **(b)** show, respectively. The unused causal relationship in (b) is annotated in pink.

∙ KDB (*K*-dependence classifier). The probability of each attribute value in KDB is conditioned by the class and other*K* attributes. The KDB algorithm adopts a greedy strategy to identify the graphical structure of the resulting classifier. KDB also achieves the weights of the relationship between attributes by computing *CMI*s that can be illustrated in a matrix. Figure 3 demonstrates the format of the matrix and an example of KDB with four predictive attributes. KDB is guided by a rigid order that is obtained by applying mutual information between predictive attributes and class variables. Mutual information does not consider the interaction among predictive attributes. This marginal knowledge may result in suboptimal order. Without loss of generality, the attribute order is assumed to be {*X*_1_,⋯,*X*_4_} by comparing mutual information. Figure 4(a) indicates the corresponding network structure of KDB when *K*=2, and the causal relationships are depicted in Figure 4(b). Although the causal relationship between *X*_2_ and *X*_1_ is the weakest, the latter is selected as the parent attribute of the former. By contrast, the strong causal relationship between *X*_4_ and *X*_1_ is neglected.

### The FKBN Algorithm

By considering more attributes as possible parent attributes, prediction performance will be improved because the chain rule is approximately achieved. FKBN applies greedy search in *CMI* space to represent the strongest causal relationships and to retain the privileges of TAN and KDB (i.e., global optimisation and higher dependency representation). On the basis of this condition, FKBN adds high dependencies at arbitrary points (values of *K*) along the attribute dependence spectrum such as KDB. The newly added arc corresponds to the strongest relationship that is not implicated in the existing tree structure. The direction of each arc should point outward to ensure the characteristic of the directed acyclic graph. In this research, we also use *CMI* to measure the weight of the relationship between attributes. Assuming that {*X*_1_,*X*_2_,⋯,*X*_*n*_} are *n* attributes and *C* is the class variable, the learning algorithm of FKBN is depicted as follows:

**Figure:**
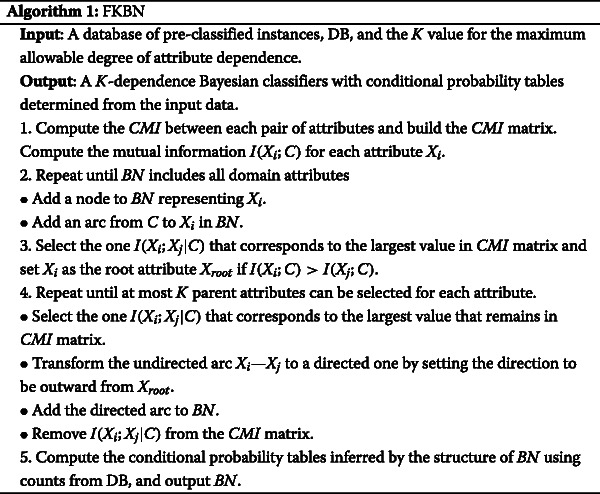

**Figure 5:**
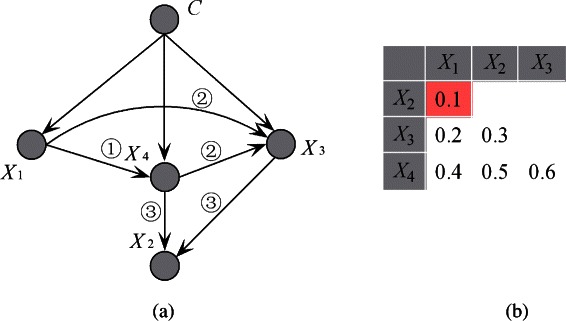
The *K*-dependence relationships among attributes inferred from FKBN and an example of *CMI* matrix are as **(a)** and **(b)** show, respectively. The unused causal relationship in **(b)** is annotated in pink.

In the above description of the algorithm, Step 4 requires the selection of most *K* parents for each attribute. We set *K*=2 in the following discussion. When *K*=2, Figure 5(a) shows the network structure of FKBN corresponding to the *CMI* matrix shown in Figure 3. The causal relationships in this case are depicted in Figure 5(b). All strong causal relationships are implicated in the final network structure.

The experimental research has been performed with the approval of the ethics committee of JiLin University of China.

## Software and programs

The following algorithms are compared:

· NB, standard Naive Bayes.

· TAN[20], Tree-augmented Naive Bayes applying incremental learning.

· KDB, standard *K*-dependence Bayesian classifier.

The experimental system is implemented in C++. The missing values for the qualitative and quantitative attributes are replaced with modes and means from the training data, respectively. For each benchmark data set, numeric attributes are discretised by using MDL discretisation[21]. Base probability estimates *P*(*c*),*P*(*c*,*x*_*i*_) and *P*(*c*,*x*_*i*_,*x*_*j*_) are smoothed by using the Laplace estimate:

(3) $$ \begin{cases} &\hat{P}(c)=\frac{F(c) + 1}{K + k}\\ &\hat{P}(c, x_{i})=\frac{F(c, x_{i}) + 1}{K_{i} + k_{i}}\\ &\hat{P}(c, x_{i},x_{j})=\frac{F(c, x_{i},x_{j}) + 1}{K_{ij} + k_{ij}} \end{cases}  $$

where *F*(·) is the frequency with which a combination of terms appears in the training data, *K* is the number of training instances for which the class value is known, *K*_*i*_ is the number of training instances for which both the class and attribute *X*_*i*_ are known, *K*_*ij*_ is the number of training instances for which all of the class, and attributes *X*_*i*_ and *X*_*j*_ are known. *k* is the number of attribute values of class *C*, *k*_*i*_ is the number of attribute value combinations of *C* and *X*_*i*_, and *k*_*ij*_ is the number of attribute value combinations of *C*, *X*_*j*_ and *X*_*i*_.

## Results

In machine learning, one of the standard measures of predicting the performance of trained models is zero-one loss, which is a powerful tool from sampling theory statistics used for analysing supervised learning scenarios [22]. Suppose *c* and $\hat {c}$ are the true class variable and the outcome of a learning algorithm, respectively, the zero-one loss function is construed as follows:

$$\xi(c,\hat{c})=1-\delta(c,\hat{c}), $$

where $\delta (c,\hat {c})=1$ if $\hat {c}=c$ and 0 otherwise. When the zero-one loss is lower, the prediction performance of a corresponding classifier is better. If the amount of data is satisfactorily large, the average zero-one loss can be estimated by using computer intensive resampling methods such as cross-validation. Cross-validation mimics the use of training and test sets by repeatedly training the algorithm *N* times with a fraction 1/*N* of training examples left out for testing purposes. Table 2 presents the comparative results of zero-one loss estimated by 10-fold cross-validation to accurately estimate the average performance of an algorithm.

**Table 2:** **Experimental results of zero-one loss and standard deviation**

Data set	NB	TAN	KDB	FKBN
BREAST	0.191 ±0.002	0.166 ±0.001	0.164 ±0.001	0.162 ±0.001
FEMGEN	0.203 ±0.002	0.149 ±0.002	0.131 ±0.001	0.128 ±0.001
LYMYLEUK	0.297 ±0.002	0.269 ±0.001	0.261 ±0.001	0.257 ±0.001
MALEGEN	0.220 ±0.002	0.163 ±0.002	0.169 ±0.002	0.160 ±0.002
COLRECT	0.197 ±0.002	0.180 ±0.001	0.177 ±0.001	0.175 ±0.001
URINARY	0.205 ±0.003	0.180 ±0.002	0.172 ±0.002	0.169 ±0.002
ALL-AML_Leukemia	0.290 ±0.405	0.289 ±0.421	0.290 ±0.435	0.289 ±0.429
DLBCL-Harvard	0.247 ±0.135	0.247 ±0.147	0.246 ±0.132	0.246 ±0.129
DLBCL-Stanford	0.489 ±0.289	0.489 ±0.309	0.489 ±0.317	0.489 ±0.277
Lung Cancer-Michigan	0.104 ±0.102	0.103 ±0.089	0.104 ±0.110	0.104 ±0.107
MLL_Leukemia	0.649 ±0.257	0.648 ±0.261	0.649 ±0.248	0.646 ±0.249
ProstateCancer	0.490 ±0.113	0.490 ±0.110	0.491 ±0.107	0.489 ±0.116

The first six data sets are selected from the SEER database, the next six data sets are selected from Kent Ridge Bio-medical (KRBM) repository.

Friedman proposed a non-parametric measure [23], Friedman test (*FT*), which ranks the algorithms for each data set separately by comparing zero-one loss. The best performing algorithm getting the rank of 1, the second best rank 2, ⋯. In case of ties, average ranks are assigned. Let ${r^{j}_{i}}$ be the rank of the *j*-th of *k* algorithms on the *i*-th of*N* data sets. *FT* compares the average ranks of algorithms, $R_{j} = \frac {1}{N}\sum _{i} {r^{j}_{i}}$. *FT* helps to compare and evaluate the overall prediction performance of different learning algorithms when dealing with numerous data sets. A difference is considered to be significant when the outcome of a two-tailed binomial sign test is less than 0.05. The experimental results of *FT* are shown in Table 3. By comparing average *FT* we can see that, the order of these algorithms is {FKBN, KDB, TAN, NB}.

**Table 3:** **Experimental results of Friedman test**

Data set	NB	TAN	KDB	FKBN
BREAST	4.0	2.0	2.0	2.0
FEMGEN	4.0	3.0	1.5	1.5
LYMYLEUK	4.0	2.0	2.0	2.0
MALEGEN	4.0	1.5	3.0	1.5
COLRECT	4.0	2.0	2.0	2.0
URINARY	4.0	3.0	1.5	1.5
ALL-AML_Leukemia	2.5	2.5	2.5	2.5
DLBCL-Harvard	2.5	2.5	2.5	2.5
DLBCL-Stanford	2.5	2.5	2.5	2.5
LungCancer-Michigan	2.5	2.5	2.5	2.5
MLL_Leukemia	2.5	2.5	2.5	2.5
ProstateCancer	2.5	2.5	2.5	2.5
**Average**	3.3	2.4	2.3	2.1

A difference is considered to be significant when the outcome of a two-tailed binomial sign test is less than 0.05.

The prediction superiority of FKBN to the other classifiers is remarkably obvious especially when dealing with large data sets. The main reason for such a condition may be the case that, when data size is large enough for probability estimation and relational dependency representation, the credible conditional dependencies among attributes extracted based on information theory play a key role in prediction. By generating a maximal spanning tree, TAN can achieve a trade-off between the model and computational complexity. Therefore, although TAN is restricted to have at most one parent node for each predictive attribute, its structure is more reasonable than NB and can relatively exhibit relationship between attributes. FKBN further relaxes the assumption by allowing at least two attributes to be parents and to increase the robustness of the final model. Furthermore, FKBN can fully extract the causal relationship between attributes by applying a dynamic searching strategy in the early building stage to identify the optimal attribute order. In this event, the final model is significantly flexible and credible.

From the viewpoint of dependency complexity, NB expresses zero-dependence because no dependency relationship exists between attributes. Similarly, TAN is a one-dependence based-Bayesian classifier. KDB and FKBN are two-dependence based-Bayesian classifiers. The KRBM data sets are exceedingly small; thus, the conditional dependencies measured by the *CMI* are weak. Accordingly, all high-dependence Bayesian classifiers (e.g., TAN, KDB or FKBN) degenerate to be NB. The results of zero-one loss reveal that they perform almost the same when dealing with KRBM data sets.

## Discussion

Breast cancer is the second leading cancer responsible for the highest mortality rate among women. Early detection and diagnosis are proven to be the only means of curbing this disease and of reducing its mortality rate. Physicians must have access to a smart system for predicting this illness on time before it is too late to be treated. Predicting the outcome of cancer and detecting dependencies among clinical variables or attributes play a pivotal role in cancer diagnosis and therapy. Over the past decades, many data mining studies have tried to predict the five-year survival rate of breast cancer patients. However, even the most accurate predictive forecasts have limited value unless they can also provide clear action procedures to induce the desired results.

**Figure 6:**
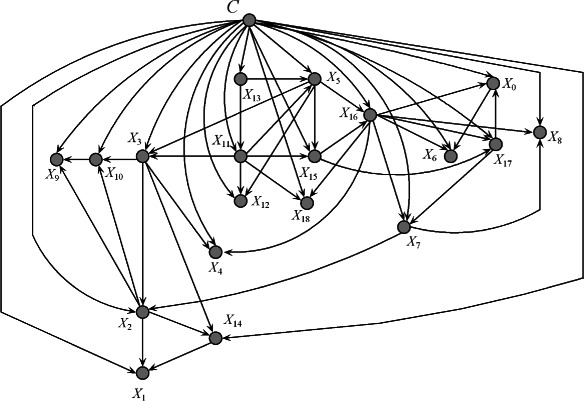
The FKBN network structure corresponding to SEER data set. Attributes {*X*_0_,*X*_1_,⋯,*X*_18_,*C*} correspond to clinical variables *Race, Sex, Age, Primary-Site, Histologic-Type, Grade, EOD-Tumor-Size, EOD-Extension, EOD-Lymph-Node-Involve, Regional-Nodes-Positive, Regional-Nodes-Examined, Rx-Summ-Surg-Prim-Site, Rx-Summ-Sur-Oth-Reg, Rx-Summ-Surg-/-Rad-Seq, CS-Schema-v0203, SEER-historic-stage-A, SEER-Summary-Stage-1977, Number-of-primaries, First-malignant-primary-indicator* and *Class*, respectively.

**Figure 7:**
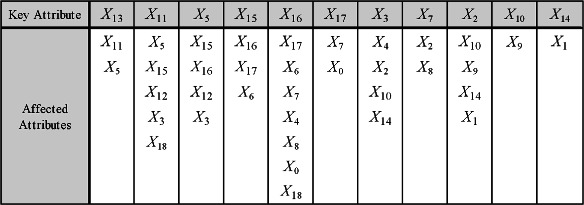
The causal relationships inferred from breast cancer data set of SEER database. Attributes {*X*_0_,*X*_1_,⋯,*X*_18_} correspond to clinical variables *Race, Sex, Age, Primary-Site, Histologic-Type, Grade, EOD-Tumor-Size, EOD-Extension, EOD-Lymph-Node-Involve, Regional-Nodes-Positive, Regional-Nodes-Examined, Rx-Summ-Surg-Prim-Site, Rx-Summ-Sur-Oth-Reg, Rx-Summ-Surg-/-Rad-Seq, CS-Schema-v0203, SEER-historic-stage-A, SEER-Summary-Stage-1977, Number-of-primaries* and *First-malignant-primary-indicator*, respectively.

**Figure 8:**
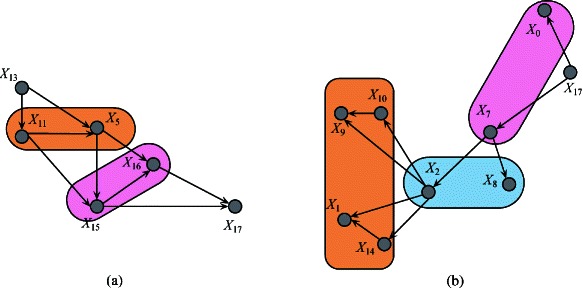
The local independent and dependent network structure of attribute *X*_17_ on SEER data set are as **(a)** and **(b)** show, respectively. Attributes {*X*_0_,*X*_1_,*X*_2_,*X*_5_,*X*_7_,*X*_8_,*X*_9_,*X*_10_,*X*_11_,*X*_13_,*X*_14_,*X*_15_,*X*_16_,*X*_17_} correspond to clinical variables *Race, Sex, Age, Grade, EOD-Extension, EOD-Lymph-Node-Involve, Regional-Nodes-Positive, Regional-Nodes-Examined, Rx-Summ-Surg-Prim-Site, Rx-Summ-Surg-/-Rad-Seq, CS-Schema-v0203, SEER-historic-stage-A, SEER-Summary-Stage-1977, Number-of-primaries* and *First-malignant-primary-indicator*, respectively.

To clarify the FKBN algorithm more clearly, we also choose 20 attributes as described in [24] from breast cancer data set in SEER database. The detailed information of the selected attributes is shown in Table 4 and is employed to develop the Bayesian model. Cause-specific death prediction is used as the class label. The graph model of the predictive attributes in the breast cancer data set (Figure 6) is generated on the basis of the results of the FKBN analysis. Figure 6 demonstrates that the attributes *X*_*key*_={*X*_13_,*X*_11_,*X*_5_,*X*_15_,*X*_16_,*X*_17_,*X*_3_,*X*_7_,*X*_2_,*X*_10_,*X*_14_}, which correspond to {*Rx-SummSurg-/-Rad-Seq, Rx-SummSurg-Prim-Site, Grade, SEER-historic-stage-A, SEER-Summary-Stage-1977, Number-of-primaries, Primary-Site, EOD-Extension, Age-at-diagnosis, Regional-Nodes-Examined and CS-Schema-v0203*}, play key roles for prediction. These attributes have the same characteristics (i.e., they are affected by other attributes and also act on other attributes). The other attributes are not parents of any other attributes and play a secondary role. For example, attribute *X*_1_, i.e. *Sex*, is dependent on *X*_2_ (*Age-at-diagnosis*) and *X*_14_ (*CS-Schema-v0203*). The causal relationships among these attributes are summarised in Figure 7. From the viewpoint of medical diagnosis, the key attributes should be considered first. Subsequently, the non-key attributes, which are dependent on key attributes, should then be extensively analysed. For example, the local dependent and independent structures of *X*_17_ are shown in Figure 8. Attribute *X*_17_ is directly dependent on attributes {*X*_16_,*X*_15_} (Figure 8(a)), which are dependent on {*X*_11_,*X*_5_}. In this case, *X*_13_ is the final cause. Doctors can follow this order to lower the time cost for diagnosis and expenditure on unnecessary physical examination. On the other hand, Figure 8(b) demonstrates that the attributes {*X*_0_,*X*_7_} are directly dependent on *X*_17_ and their values may be affected by *X*_17_. Furthermore, {*X*_2_,*X*_8_} and then {*X*_9_,*X*_10_,*X*_1_,*X*_14_} will be affected by *X*_17_ indirectly. In the following discussion, we will clarify Figure 6 with respect to several common relationships *Sex*/*Age*, *Race*/*SEER-Summary-Stage* and *Tumor Size*/*Race*, respectively.

**Table 4:** **Attributes available for analysis**

Attribute	Type	Explanation	Corresponding
			symbol in
			Figures 6, 7, 8
Race/ethnicity	Categorical	22 races	*X*_0_
Sex	Binary	Female/male	*X*_1_
Age-at-diagnosis	Numeric	Years	*X*_2_
Primary-Site	Categorical	Eleven sites	*X*_3_
Histologic-Type-ICD-O-3	Categorical	International Prediction of Diseases for Oncology Third Revision	*X*_4_
Grade	Categorical	Tumor grade	*X*_5_
EOD-Tumor-Size	Numeric	Size of primary tumor	*X*_6_
EOD-Extension	Categorical	Invasive extension of primary tumor	*X*_7_
EOD-Lymph-Node-Involve	Categorical	Extension of lymph node involvement	*X*_8_
Regional-Nodes-Positive	Numeric	No. of positive regional lymph nodes	*X*_9_
Regional-Nodes-Examined	Numeric	No. of regional lymph nodes examined	*X*_10_
Rx-Summ-Surg-Prim-Site	Categorical	Extension of surgery	*X*_11_
Rx-Summ-Sur-Oth-Reg/Dis	Categorical	Surgery of other regional site(s), distant site(s), or distant lymph node(s)	*X*_12_
Rx-Summ-Surg-/-Rad-Seq	Categorical	Prior to/after surgery/both	*X*_13_
CS-Schema-v0203	Categorical	CS information collected based on site and histology	*X*_14_
SEER-historic-stage-A	Categorical	A stage system coded by SEER	*X*_15_
SEER-Summary-Stage-1977	Categorical	A stage system coded by SEER	*X*_16_
Number-of-primaries	Numeric	Number of primaries	*X*_17_
First-malignant-primary-indicator	Binary	Yes/no	*X*_18_
Class	Categorical	SEER cause-specific death prediction	*C*

***Sex/Age:*** Approximately 343,919 cases of breast cancer were expected to be diagnosed in women, along with 2,398 cases in men. Figure 6 indicates that a direct relationship exists between the *Sex* (*X*_1_) and *Age* (*X*_2_). Accordingly, *Age* should be considered a complementary factor of *Sex* for cancer diagnosis. A statistical analysis of the breast cancer data set reveals that incidence rate begins to increase when the woman is 40 years old and reaches its maximum between 54 and 68 years old. This event may be due to tumours diagnosed at younger ages being more aggressive and/or less responsive to treatment. The age of 40 is also a turning point for mortality rate. The mortality rate of women decreases when their age increases from 0 to 40. The mortality rate will then remain stable when the age is between 40 and 70. When the age increases from 70 to 110, the mortality rate will increase and reach its maximum. Older patients may reflect lower rates of screening, detection of cancers via mammography and/or incomplete detection. In this research, the determined median age at the time of breast cancer diagnosis was 60.78. This finding implies that half of women who developed breast cancer were 61 years old or younger at the time of diagnosis. Meanwhile, similar to female breast cancer, the cause-specific mortality rates of male breast cancer generally increase with age. Given the infrequency of male breast cancer, which accounts for less than 1% of all breast cancer cases, remarkably less confident information can be inferred to predict the outcome of such a disease.

***Race/SEER-Summary-Stage:*** The American Cancer Society has determined notable differences in breast cancer mortality rates between different states across various socioeconomic strata and between different racial/ethnic groups. The statistical analysis of breast cancer data set illustrates that Caucasian women are more likely to develop breast cancer. In fact, Caucasian women account for 84.92% of all breast cancer cases and African-American women account for only 10.23% of all cases. However, a substantial racial gap can be observed in mortality rate. In particular, the findings of this research indicated that the morality rate for Caucasian and African-American women were 7.68% and 13.47%, respectively. Figure 6 specifies that a causal relationship exists between *Race* (*X*_0_) and *SEER-Summary-Stage* (*X*_16_). This survival disparity is attributed to the latter stage of detection among African-American women, who have the highest morality rate among any racial or ethnic group. The presence of additional illnesses, lack of health insurance and disparities in receipt of treatment probably contribute to the differences in breast cancer survival.

***Tumor Size/Race:*** The incidence rates of breast cancer by tumour size greatly differ. American women are less likely to be diagnosed with middle-sized tumours and more likely to be diagnosed with larger (>5.0 cm) or smaller tumours (<1.5 cm). Mortality rate increases with increasing tumour. The mortality rate corresponding to smaller tumours is 6.02%, and the mortality rate corresponding to middle-sized tumours and larger tumours are 9.86% and 13.21%, respectively. Figure 6 demonstrates that a causal relationship exists between*Tumour Size* (*X*_6_),*Race* (*X*_0_) and *SEER-Summary-Stage* (*X*_16_). For smaller tumours, the incidence rate is 17.72% among Caucasian women, but 11.84% among African-American women. The incidence rate for larger tumours (>5.0 cm) is 8.74% among Caucasian women, but 10.95% among African-American women. This incidence disparity can also be attributed to both later cancer stages at detection and poorer stage-specific survival among African-American women. Poverty, low education and unequal access to medical care are associated with low breast cancer incidence.

## Conclusion

BNs can graphically describe conditional dependency among attributes and have been previously identified as computationally efficient approaches for further reducing prediction error. The proposed algorithm, namely, FKBN, offers a trade-off between probability estimation and network structure complexity. With enough instances to detect reliable dependencies among predictive attributes, the findings of this study are helpful in diagnostic practice and drug design. Possible extensions of this investigation should involve applying the novel computational framework in categorising other diseases and detecting properties that can be targeted for cancer therapy. Moreover, computational advancement will require improving the prediction accuracy of the proposed methodology by updating it to existing algorithms.
